# Antioxidant-Loaded Mesoporous Silica—An Evaluation of the Physicochemical Properties

**DOI:** 10.3390/antiox11071417

**Published:** 2022-07-21

**Authors:** Adrian Szewczyk, Joanna Brzezińska-Rojek, Justyna Ośko, Dorota Majda, Magdalena Prokopowicz, Małgorzata Grembecka

**Affiliations:** 1 Department of Physical Chemistry, Faculty of Pharmacy, Medical University of Gdańsk, Gen. J. Hallera Avenue 107, 80-416 Gdańsk, Poland; adrian.szewczyk@gumed.edu.pl; 2 Department of Bromatology, Faculty of Pharmacy, Medical University of Gdańsk, Gen. J. Hallera Avenue 107, 80-416 Gdańsk, Poland; joanna.brzezinska@gumed.edu.pl (J.B.-R.); justyna.osko@gumed.edu.pl (J.O.); 3 Faculty of Chemistry, Jagiellonian University in Kraków, Gronostajowa 2, 30-387 Kraków, Poland; dorota.majda@uj.edu.pl

**Keywords:** antioxidants, mesoporous silica, adsorption, desorption, antioxidant potential

## Abstract

The dangerous effects of oxidative stress can be alleviated by antioxidants—substances with the ability to prevent damage caused by reactive oxygen species. The adsorption of antioxidants onto nanocarriers is a well-known method that might protect them against rough environ-mental conditions. The aim of this study was to investigate the adsorption and desorption of gallic acid (GA), protocatechuic acid (PCA), chlorogenic acid (CGA), and 4-hydroxybenzoic acid (4-HBA) using commercially available mesoporous silica materials (MSMs), both parent (i.e., SBA-15 and MCM-41) and surface functionalized (i.e., SBA-NH2 and SBA-SH). The MSMs loaded with active compounds were characterized using Fourier transform infrared spectroscopy (FTIR), scanning electron microscopy with energy-dispersive X-ray spectroscopy (SEM-EDX), thermogravimetric analysis (TGA), differential scanning calorimetry (DSC), thermoporometry (TPM), and powder X-ray diffraction (XRD). High-performance liquid chromatography (HPLC-CAD) was used to evaluate the performance of the adsorption and desorption processes. The antioxidant potential was investigated using the Folin–Ciocalteu (FC) spectrophotometric method. Among the studied MSMs, the highest adsorption of GA was observed for amine-modified SBA-15 mesoporous silica. The adsorption capacity of SBA-NH2 increased in the order of PCA, 4-HBA < GA < CGA. Different desorption effectiveness levels of the adsorbed compounds were observed with the antioxidant capacity preserved for all investigated compounds.

## 1. Introduction

The oxidation–antioxidation balance is a physiological state in which the amount of reactive oxygen species (ROS) and their formation rate remain in equilibrium with the mechanisms of their neutralization [1]. Reactive oxygen species formed in the body provide its homeostasis and proper functioning; however, an imbalance in ROS production may result in their accumulation in excess amounts causing oxidative stress [2]. Oxidative stress disrupts cellular signaling, resulting in cell damage and increasing the risk of civilization diseases such as ischemic heart disease [3,4], cancer [5], and hypertension [6]. The dangerous effects of oxidative stress can be alleviated using antioxidants. These are substances with the ability to prevent the damage caused by ROS via different mechanisms: scavenging free radicals [2,7], inhibition of oxidative enzymes [2], or complex formation with metal ions, which prevents catalysis of free radicals’ reactions [7]. There can be distinguished enzymatic (endogenous) and nonenzymatic antioxidants [8]. Nonenzymatic antioxidants are most often vitamins [9] or secondary metabolites of plants, especially polyphenols, which are abundant in foods of plant origin [10].

Among polyphenols, particular attention has been paid to benzoic acid derivatives, such as gallic acid, protocatechuic acid, and chlorogenic acid, which have been extensively examined as antioxidants. Gallic acid (3,4,5-trihydroxybenzoic acid; GA), one of the most abundant phenolic acids in plants, is characterized by anti-inflammatory, antimicrobial, anticancer, gastroprotective, cardioprotective, and neuroprotective activities confirmed in both in vitro and in vivo studies [11]. Protocatechuic acid (3,4-dihydroxybenzoic acid; PCA) is a common compound found in the human diet; rich sources of PCA are bran, brown rice, and onion [12]. The antioxidative properties of PCA have been investigated in vivo with potential application as a chemo-preventive agent [12]. It was found that PCA inhibits chemical carcinogenesis in rodents [13], reduces the glycation-associated diabetic complications in mice [14], and exhibits anti-inflammatory and analgesic activity comparable to diclofenac sodium in both rats and mice models [15]. Chlorogenic acid (ester of caffeic acid and quinic acid; CGA) is frequently present in many different dietary sources, especially in coffee beans, potato tubers, eggplant, artichoke, and sunflower seeds [16]. Studies have indicated that CGA has antimicrobial activity against various multiple drug-resistant bacteria [17], decreases diet-induced obesity in rats (i.e., by modulating PPARα transcription [18]), and alters the level of nitric oxide, providing a hypotensive effect in rats [19]. Another promising antioxidant of benzoic acid derivatives is 4-hydroxybenzoic acid (4-HBA)—a monophenolic compound found in raspberries, strawberries, triticale, and oats [20]. The high antioxidative potential of 4-HBA has been confirmed in various studies [21]. Moreover, esters of 4-HBA, known as parabens, exhibit high antimicrobial activities against both Gram-positive and Gram-negative bacteria [22,23].

Unfortunately, polyphenols are usually characterized by relatively low bioavailability from plants [24,25] and limited stability, especially in aqueous solutions. Polyphenols are sensitive to oxidizers, heat, pH, light, and enzymes due to the unsaturated bonds present in their structure [26,27]. Moreover, their decreased activity might be observed, since they are unstable during food manufacturing, transportation, and storage [28]. Therefore, the development of procedures that allow for the obtainment of plant extracts with the preservation of antioxidant substances, in an unchanged form and at a suitable level of biological activity, has become extremely important. 

The adsorption of antioxidants onto nanocarriers is a well-known method that might protect them against rough environmental conditions [29,30]. It has been proved that antioxidants encapsulated into different matrices are characterized by prolonged shelf-life due to the enhanced stability inside the pores [31]. Moreover, antioxidant-loaded nanocarriers have also been examined in terms of nutraceuticals to modify their physicochemical properties, such as taste or flavor, or to improve both the release profiles and biostability together with bioavailability [32]. Polyphenolic extracts from grape pomace [33] or olive mill wastewaters [34] have already been adsorbed onto mesoporous silica and investigated in terms of physicochemical properties, antioxidative properties, and cytotoxicity.

Among nanocarriers, particular attention has been paid to mesoporous silica materials (MSMs). The MSMs are obtained using the soft-templating sol-gel method and are characterized by unique properties such as ordered porous arrangement, uniform pore size, high surface area and adsorption capacity, and chemical and thermal stability [35]. Moreover, the simple modification of MSMs’ surfaces with various functional groups, both polar and nonpolar, allows for the adsorption of multiple different types of molecules, especially drugs [36]. In vivo, MSMs undergo gradual resorption into orthosilicic acid; thus, they are considered biocompatible and nontoxic [37]. The United States Food and Drug Administration (FDA) classified silica as “generally recognized as safe”. Presently, MSMs are being investigated as tumor-targeted imaging agents in human clinical studies approved by the FDA [38]. 

Nowadays, studies increasingly focus on the adsorption of different polyphenols onto the most popular types of MSMs: SBA-15 and MCM-41 [39]. Cotea et al. proposed SBA-15 as an adsorbent for bioactive polyphenols from red wine [40], whereas adsorption and release of resveratrol for both SBA-15 and MCM-41 MSMs were reported by Ionita et al. [41]. In the case of polyphenolic extracts, the successful loading of a hydroalcoholic extract from grape pomace into mesopores of MCM-41 was achieved by Brezoiu et al. [33]. Additionally, antimicrobial activity against Gram-positive bacteria of black chokeberry extract loaded into MCM-41 silicas was proved by Buda et al. [42]. Moreover, MSMs are considered as novel nanocarriers for GA. MCM-41 silica was proposed as a delivery matrix of pH-sensitive GA [43]; Lewandowski et al. covalently conjugated GA to SBA-15 mesoporous silica and analyzed the cytotoxicity of such a complex [44]. Iraji et al. confirmed the prolonged release of GA-loaded MCM-41 MSMs together with their killing potency against the breast cancer cell line (MCF-7) [45], whereas Rashidi et al. observed a cytocompatibility of GA-loaded MCM-41 silica with Caco-2 cells [46]. 

In the studies presented above, either synthesis or modification of MSMs was performed manually in the investigators’ lab. Herein, we provide a comprehensive study of benzoic acid derivatives’ adsorption–desorption onto the commercially available MSMs. To the best of our knowledge, the studies on the adsorption of GA, PCA, CGA, and 4-HBA onto commercially available MSMs: MCM-41, SBA-15, SBA-15 amine functionalized (SBA-NH2), and SBA-15 thiol functionalized (SBA-SH) have not been investigated. It was found that the number of hydroxyl groups significantly influenced the efficiency of the adsorption process. On the other hand, the analysis of desorption showed the different effectiveness levels of this process for the adsorbed substances. Based on the Folin–Ciocalteu method, the antioxidant capacity was preserved for all of the compounds tested.

## 2. Materials and Methods

### 2.1. Materials 

The mesoporous silica materials, MCM-41 (<150 μm particle size, 2.1–2.7 nm pore size, hexagonal pore morphology), SBA-15 (<150 μm particle size, 6 nm pore size, hexagonal pore morphology), SBA-NH2 (<150 μm particle size, 6 nm pore size, amine functionalized), and SBA-SH (<150 μm particle size, 6 nm pore size, thiol functionalized), were purchased from Merck and used as received. All antioxidants (i.e., GA, PCA, CGA, and 4-HBA) were of analytical grade (HPLC purity > 97.0%) and purchased from Sigma-Aldrich. All aqueous solutions were prepared using ultrapure water (18.2 MΩ·cm, Milipore Simplicity System, Billerica, MA, USA). HPLC grade chemicals were obtained from J.T. Baker (Mallinckrodt Baker, Phillipsburg, NJ, USA). Reagents for the Folin–Ciocalteu method were as follows: ethanol 96% (*v/v*) (Emsure, Darmstadt, Germany), anhydrous sodium carbonate (purity > 99.5%, Chempur, Piekary Śląskie, Poland), Folin–Ciocalteu reagent (analytical grade, Chempur, Piekary Śląskie, Poland).

### 2.2. Adsorption of Antioxidants onto MSMs

#### 2.2.1. Adsorption of GA onto MCM-41, SBA-15, SBA-NH2, and SBA-SH

First, GA, as a model compound, was adsorbed onto MCM-41, SBA-15, SBA-NH2, and SBA-SH to select the material with the highest adsorption efficiency. A concentrated solution of GA in a purified water–ethanol mixture (6:4 *v/v*) was obtained (11.4 mg/mL, which corresponded to 90% of the GA solubility in the purified water–ethanol mixture). Next, 200 mg of each MSMs was suspended in 5 mL of GA-concentrated solution and left for 24 h under stirring conditions (300 rpm, room temperature) to provide an adsorption equilibrium state. After 24 h, the suspensions were centrifuged (1500 rpm, 15 min) and the supernatant was collected. The stability of antioxidants during the 24 h of incubation was confirmed in the preliminary studies. The GA concentration in the solution before and after the adsorption was measured using the preliminarily validated HPLC-CAD method according to conditions mentioned in Section 2.3. Briefly, 0.109 mL of GA solution was replenished in a volumetric flask to 25 mL with water and 0.1% formic acid. Then, 1 mL of diluted sample was placed in a vial and analyzed. The experiment was performed in three replicates, and the adsorption efficiency of GA onto MCM-41, SBA-15, SBA-NH2, and SBA-SH materials is expressed as the mean value ± standard deviation (SD). The adsorption efficiency together with the amount of GA adsorbed onto each material were calculated using Equations (1) and (2), respectively:

(1) $$\%\mathrm{Ads}=\frac{C_{0}\;{-\;C}_{e}}{C_{0}}\;\times \;100\%$$

(2) $$m_{\mathrm{Ads}}=\frac{C_{0}{\;-\;C}_{e}}{m}\times V$$

where %Ads—adsorption efficiency (%); C_0_—initial concentration of GA (mg/mL); C_e_—concentration of GA at an equilibrium state (mg/mL); V—volume of the GA solution (mL); m_Ads_—mass of GA adsorbed onto MSM at an equilibrium state (mg); m—mass of MSM used (g).

#### 2.2.2. Adsorption of PCA, CGA, and 4-HBA onto SBA-NH2

The SBA-NH2 type of MSM, characterized by the highest adsorption efficiency of GA, was used as an adsorbent of additional benzoic acid derivatives: PCA, CGA, and 4-HBA. Both the procedure and conditions of adsorption were analogical to the adsorption of GA. The concentrated PCA, CGA, and 4-HBA solutions used in the adsorption procedure corresponded to 90% solubility in the purified water–ethanol mixtures (6:4 *v/v*), which amounted to 14.4, 35.3, and 31.9 mg/mL, respectively. All obtained antioxidant-loaded MSMs were frozen (−30 °C) for 24 h and then lyophilized (Alpha 1–4 LD plus freeze dryer; −42 °C, 0.1 mbar, 24 h, and 20 min of drying off at −50 °C, 0.02 mbar), homogenized in mortar, and stored under −30 °C prior to the further analyses.

### 2.3. Adsorption Efficiency—HPLC Analysis

The adsorption efficiency of the antioxidants adsorbed onto the MSMs was examined using chromatographic analyses performed on a high-performance liquid chromatograph Ultimate 3000 system (HPLC-CAD, Dionex, Germering, Germany) coupled with a Corona CAD detector (ESA, Part No. 70–6186A; Serial No. CO-0602, Chelmsford, MA, USA, 2007). Data processing was carried out with Chromeleon™ 6.8 Chromatography Data System (CDS) Software (Dionex, Germering, Germany), and the nitrogen gas flow rate was regulated automatically and monitored by the CAD device. Gas (35 psi) was supplied by nitrogen generator Alize 7.1.3 (F-DGSi, Paris, France). Chromatographic separation was achieved using a Hypersil Gold (Thermo Fisher Scientific, Waltham, MA, USA), 5 μm (250 × 4.6 mm) column with a guard pre-column. The mobile phase (A + B) was composed of: A—water with 0.1% formic acid; B—acetonitrile. A gradient run was applied as follows: 0–5 min (10% B); 5–10 min (10–20% B); 10–12 min (20% B); 12–15 min (20–45% B); 15–18 min (45% B); 18–10 min (45–10% B); 20–22 min (10% B). Each run was completed within 22 min with a mobile phase flow at 1.0 mL/min. The column, as well as the autosampler temperature, were maintained at 15 °C. The injection volume was 20 μL. Detector (CAD) settings were as follows: 100 pA; filter—none.

The validation of the HPLC-CAD method was carried out by evaluating the linearity range, precision, repeatability, limit of detection (LOD), limit of quantification (LOQ), and accuracy. In order to verify the linearity range, five curves (six-fold repetition) were prepared for all antioxidants investigated. The plotted curves were based on 10 concentration points in the range from 0.25 to 100 μg/mL. The determination factor R^2^ was higher than 0.996 (R^2^ = 0.9963–0.9995). Both LOD and LOQ were calculated, and the results were in the range of 0.04–0.64 and 0.11–1.92 μg/mL, respectively. The standard addition method was applied to determine the accuracy and precision. The repeatability was checked by analyzing the same working standard for two consecutive days, and the results were satisfactory for all antioxidants investigated (relative SD < 4.54%).

### 2.4. Desorption of Antioxidants from MSMs

Fifty milligrams of the homogenized samples of antioxidant-loaded MSMs were suspended in 3 mL of the purified water–ethanol mixture (6:4 *v/v*) and vigorously stirred (300 rpm) for 24 h at room temperature. Next, suspensions were centrifuged at 1500 rpm for 15 min. Supernatants were collected and analyzed by HPLC-CAD using the conditions mentioned in Section 2.3. The dilution of the supernatants was adjusted using water and 0.1% FA. The percentage of antioxidants desorbed from MSMs (%Des) with a corresponding mass (m_Des_) was calculated using Equations (3) and (4), respectively:

(3) $$\%\mathrm{Des}=\frac{C_{\mathrm{Des}}\;{\times \;V}_{\mathrm{Des}}}{m_{\mathrm{Ads}}}\;\times \;100\%$$

(4) $$m_{\mathrm{Des}}{=C}_{\mathrm{Des}}\;{\times \;V}_{\mathrm{Des}}$$

where %Des—percentage of desorption; C_Des_—concentration of antioxidant in solution after desorption (mg/mL); V_Des_—volume of solution (mL); m_Ads_—calculated mass of adsorbed antioxidant onto the MSM; m_Des_—calculated mass of antioxidant desorbed from the MSM.

### 2.5. Antioxidant Capacity Assay

The ability of antioxidants desorbed from MSMs to reduce phosphomolybdic/phosphotungstic acid reagent was determined using the Folin–Ciocalteu (FC) method [47]. In this method, the antioxidant capacity of phenolic compounds was based on the redox reaction in which hydroxyl groups were oxidized by a molybdotungstophosphoric heteropolyanion reagent with simultaneous formation of blue complexes. According to the FC protocol, the measurements were performed using a UV-Vis spectrophotometer (Genesys 10S, Thermo Fisher Scientific, Waltham, MA, USA). Next, 0.109 mL of the supernatants collected after the desorption procedure was taken and made up to 25 mL with water in a volumetric flask (solution D1), and 0.645 mL of solution D1 was mixed with 0.355 mL of water in a test tube. Then, 5 mL of FC reagent (FCR) was added, and after 3 min, 10 mL of saturated sodium carbonate solution (150 g/L) was added. The contents of the test tube were thoroughly mixed after each portion of the reagents using a vortex (Lab dancer, VWR, Gdańsk, Poland). The mixture was incubated in the absence of light for 30 min. Finally, absorbance was measured at a wavelength of 760 nm. Each measurement was performed in triplicate. There were prepared individual calibration curves (six-fold repetition) for GA, PCA, CGA, and 4-HBA as the standards. All the curves were linear (R^2^ = 0.9937–0.9993) within the given range (i.e., 0.1–10.0 μg/mL for GA, PCA, and CGA and 0.1–25.0 μg/mL for 4-HBA), which shows that the results obtained were directly proportional to the content of the substance. The precision and accuracy of the Folin-Ciocalteu method were determined by the standard addition method using a GA standard solution. The accuracy of the method ranged between 95 and 115%, precision between 0.78 and 1.67%. The LOD and LOQ parameters for GA, PCA, CGA, and 4-HBA were 0.012 and 0.036 μg/mL, 0.203 and 0.608 μg/mL, 0.070 and 0.211 μg/mL, and 0.049 and 0.149 μg/mL, respectively.

The antioxidant capacity (AC) was expressed as a ratio between the amount of desorbed antioxidants with preserved antioxidant capacity (determined using the FC method) and the total amount of desorbed antioxidants (determined using the HPLC method) using Equation (5):

(5) $$\mathrm{AC}=\frac{\;\%\mathrm{Des}\;(\mathrm{FC})}{\%\mathrm{Des}\;(\mathrm{HPLC})}$$

### 2.6. Physicochemical Analysis of Antioxidant-Loaded MSMs

#### 2.6.1. SEM-EDX Analysis

The surface morphology of GA-loaded MSMs was examined using scanning electron microscopy with energy-dispersive X-ray spectroscopy (SEM-EDX) analysis (Quanta 3D FEG, Lublin, Poland). Three individual 5 mg samples of MCM-41-GA, SBA-15-GA, SBA-NH2-GA, and SBA-SH-GA powders were investigated independently. Three random sites of interest were chosen each time. All prepared samples were coated with a 10 nm gold layer and analyzed using 5.0–20.0 kV operating voltage. 

#### 2.6.2. XRD Analysis

Wide-angle powder X-ray diffraction (XRD) data for both the PCA, CGA, and 4-HBA reference samples and the antioxidant-loaded SBA-NH2 materials were recorded with an Empyrean PANalytical diffractometer (Malvern, Lublin, Poland) using CuKα radiation (40 kV and 25 mA) at a scanning rate of 1 deg/min with a step width of 0.02 in the 2θ range of 6–70. Prior to analysis, 5 mg of powder was homogenized in a mortar.

#### 2.6.3. FTIR Analysis

The Fourier transform infrared spectroscopy (FTIR) spectra of both the antioxidant and MSM reference samples together with the antioxidant-loaded MSMs were recorded on the FTIR-4700 model (Jasco, Gdansk, Poland) using the KBr tablet technique. Each 1 mg of the sample was mixed with 100 mg of KBr, compressed, and analyzed in the range of 4000–400 cm^−1^.

#### 2.6.4. Thermal Analyses

Differential scanning calorimetry (DSC) and thermoporometry (TPM) measurements were performed with the use of the DSC 821e Mettler Toledo apparatus (Mettler Toledo, Cracow, Poland). In the DSC experiments, the samples were heated from 25 to 600 °C at a heating rate of 10 °C/min in an argon atmosphere (60 cm^3^/min). 

TPM was carried out with the use of water as a probe liquid. Before the TPM experiment, the samples were placed in aluminum pans, water was added, and the pans were sealed with lids. The samples were quenched to −40 °C with a cooling rate of 10 °C/min and then heated at a rate of 2 °C/min to 10 °C. After the experiment, a small hole was made in the lids of the crucibles, and the samples were heated to 200 °C to evaporate the liquid and the samples’ masses were measured. Pore size distribution was determined from the solid to liquid DSC profiles. The melting point depression was obtained relative to the excess phase so that each experiment was internally calibrated for temperature [48].

Thermogravimetric analysis (TGA) was performed on TGA/SDTA 851e Mettler Toledo equipment (Mettler Toledo, Cracow, Poland). In the experiments, the samples were heated from 25 to 600 °C at a heating rate of 10 °C/min in an argon atmosphere (60 cm^3^/min).

## 3. Results

### 3.1. Adsorption of Antioxidants onto MSMs

#### 3.1.1. Adsorption of GA onto MCM-41, SBA-15, SBA-NH2, and SBA-SH

**Figure A1:**
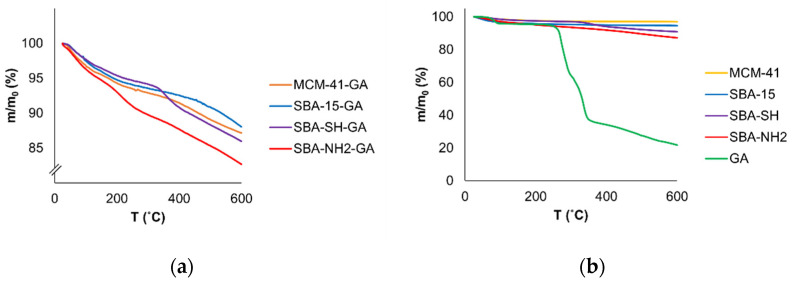
The TGA curves of (**a**) GA-loaded MSMs samples: MCM-41-GA (orange), SBA-15-GA (blue), SBA-SH-GA (purple), and SBA-NH2-GA (red); (**b**) reference samples before GA adsorption: MCM-41 (yellow), SBA-15 (blue), SBA-SH (purple), SBA-NH2 (red), GA (green).

The adsorption efficiencies (%Ads) with corresponding amounts of GA (m_Ads_) adsorbed onto four different types of MSMs are presented in Table 1. The %Ads increased in the order: SBA-15, SBA-SH < MCM-41 < SBA-NH2. The GA loading onto MSMs was also confirmed by TGA analysis, where the percentage of mass loss for the GA-loaded samples was higher compared to parent materials (Figure A1).

**Figure 1:**
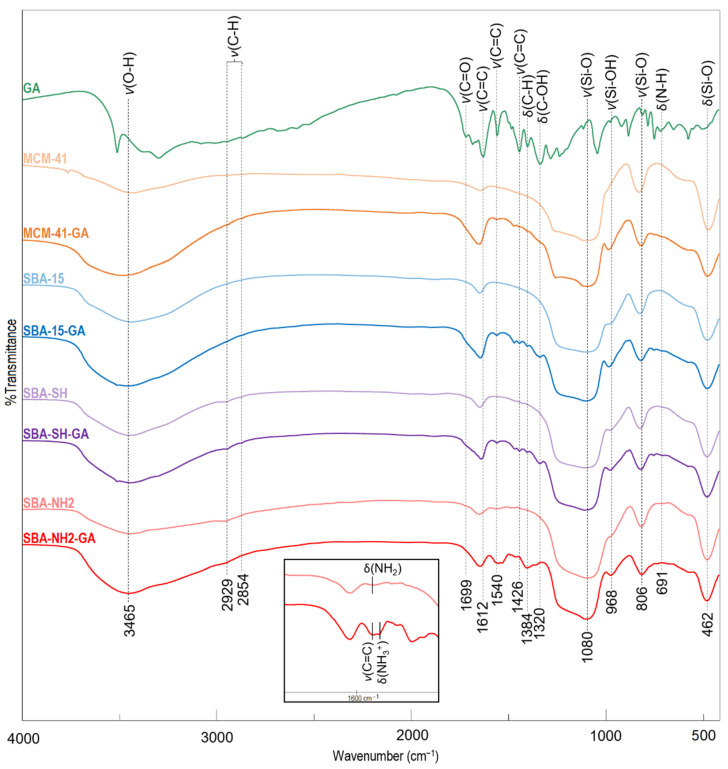
The FTIR spectra of GA-loaded MSMs together with the reference samples (types of vibrations: υ—stretching; δ—bending).

The FTIR spectra of the reference materials and GA-loaded MSMs are presented in Figure 1. In the region between 2000 and 1300 cm^−1^, the dominant bands in the spectrum of the GA reference sample were observed at 1699 cm^−1^ (C=O stretching of a carboxyl group); 1612, 1540, and 1426 cm^−1^ (C=C vibrational modes of aromatic ring); 1384 cm^−1^ (C-H stretching of aromatic ring); 1320 cm^−1^ (C-OH bending of hydroxyl groups) [49,50,51]. 

In the spectra of both unloaded and GA-loaded MSM samples, bands characteristic of silica were observed at ~3400 and ~1620 cm^−1^ (related to the H-O-H vibrations of physisorbed water); ~1080 cm^−1^ (Si-O-Si asymmetric stretching); ~965 cm^−1^ (Si-OH stretching); ~800 cm^−1^ (Si-O-Si symmetric stretching); ~462 cm^−1^ (O-Si-O deformation vibrations) [52]. Additionally, surface-modified silicas (i.e., SBA-SH and SBA-NH2) were characterized by two bands at 2900–2800 cm^−1^ derived from anchored 3-aminopropyl and 3-mercaptopropyl functional groups. For SBA-NH2, a band of N-H bending vibrations at 1540 cm^−1^ was also observed [53]. After the adsorption, the bands characteristic of GA were presented in all obtained samples, with shifts in bands characteristic of GA in the region of 1700–1300 cm^−1^. 

**Figure 2:**
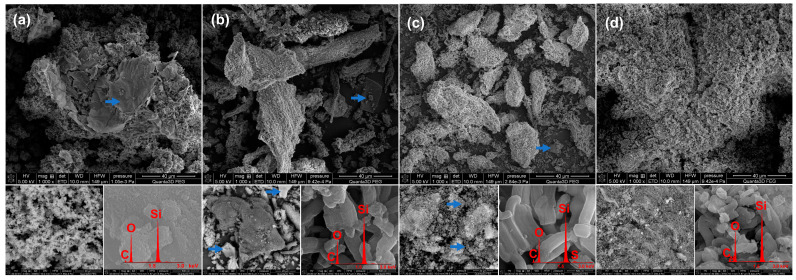
The SEM-EDX micrographs of GA-loaded MSMs samples: (**a**) MCM-41-GA; (**b**) SBA-15-GA; (**c**) SBA-SH-GA; (**d**) SBA-NH2-GA. Blue arrows represent the GA precipitates.

The SEM-EDX micrographs of GA-loaded MSMs samples are presented in Figure 2. The MCM-41-GA sample was observed in the form of agglomerated nanoparticles with spherical morphology typical for MCM-41-type MSMs [54]. The particles of GA precipitated onto MCM-41-GA samples were also observed. All GA-loaded SBA-15 samples (both parent and functionalized) exhibited the characteristic compact, elongated, rodlike morphology of the particles [55]. In the case of the SBA-15-GA and SBA-SH-GA samples, a relatively higher occurrence of precipitated GA plates covered by silica was observed, compared to SBA-NH2-GA [56].

Based on the obtained HPLC, FTIR, TGA, and SEM-EDX results, the SBA-NH2 silica was typed as an adsorbent of additional antioxidants: PCA, CGA, and 4-HBA.

#### 3.1.2. Adsorption of GA, PCA, CGA, 4-HBA, onto SBA-NH2

The adsorption efficiency with corresponding amounts of GA, PCA, CGA, and 4-HBA adsorbed onto SBA-NH2 is presented in Table 2. Based on the obtained results, the highest adsorption onto SBA-NH2 was noticed for CGA. The mass of CGA adsorbed was 2.2- and 5.4-times higher compared to GA and PCA or 4-HBA, respectively.

**Figure A2:**
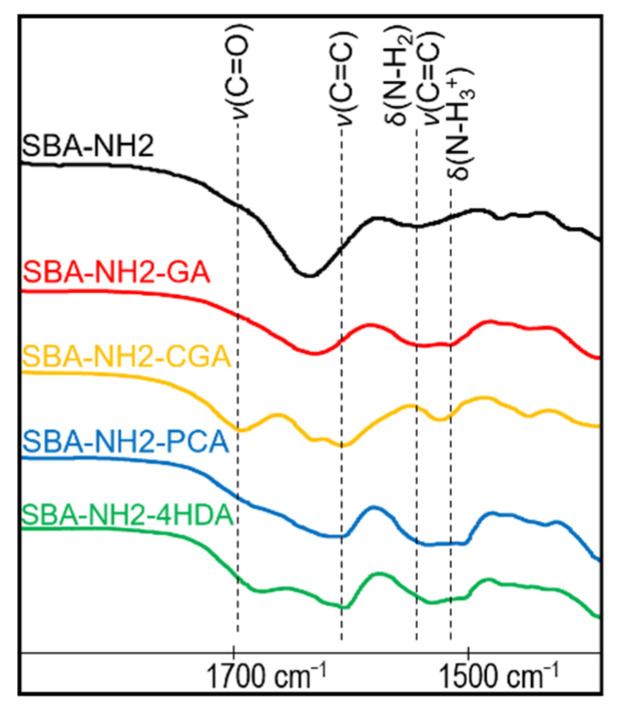
The FTIR spectra of parent SBA-NH2 and antioxidant-loaded SBA-NH2 silica materials (types of vibration: υ—stretching; δ—bending).

Moreover, a similar amount of adsorbed PCA (52.7 ± 13.5) and 4-HBA (54.5 ± 4.0 mg/g) were observed, despite their different solubility values in the adsorption medium (35.4 and 16.0 mg/mL, respectively). Similar to the adsorption of GA, shifts of bands in the region of 1700–1500 cm^−1^ were present in the FTIR spectra of all antioxidant-loaded SBA-NH2 samples (Figure A2).

**Figure 3:**
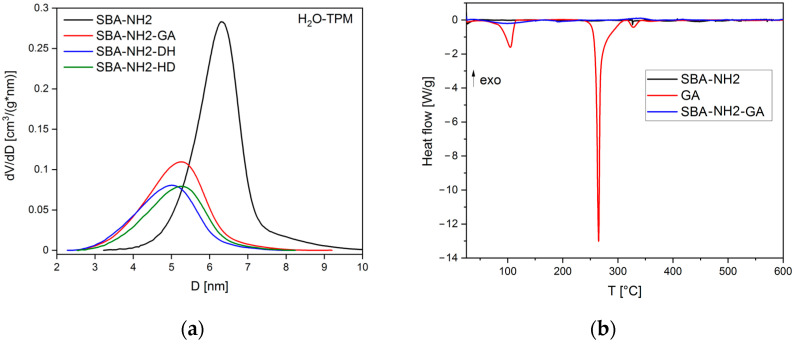
(**a**) Pore size distribution of parent and antioxidant-loaded SBA-NH2 materials based on H_2_O-TPM; (**b**) DSC thermograms of the SBA-NH2, SBA-NH2-GA, and GA reference samples.

**Figure A3:**
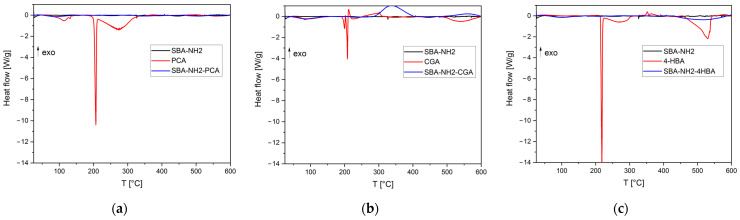
The DSC thermographs of antioxidant-loaded SBA-NH2 silica: (**a**) SBA-NH2-PCA; (**b**) SBA-NH2-CGA; (**c**) SBA-NH2-4HBA.

The results obtained from TPM confirmed the uniform pore distribution of commercially available SBA-NH2 material (Figure 3a), with a pore diameter of 6.3 nm and a pore volume of 0.41 (Table 3). The adsorption of antioxidants caused a reduction of both pore size and pore volume. In the case of the SBA-NH2-CGA sample, the porosimetric parameters were below the detection limit of TPM, which is c.a. 3 nm [48]. Examples of DSC thermographs are presented in Figure 3b. The GA reference sample was characterized by three endothermic peaks with minima at 104, 265, and 331 °C, corresponding to the evaporation of water molecules released from the monohydrate form, melting point, and residual decomposition of GA, respectively [57,58,59]. No peaks characteristic of GA were observed in the thermogram of the SBA-NH2-GA sample. The same phenomenon was observed for all investigated antioxidant-loaded SBA-NH2 materials (Figure A3). For example, the peaks characteristic of the melting points of PCA [60], CGA [61], and 4-HBA [62] at 207, 208, and 218 °C, respectively, were not present in the thermograms of antioxidant-loaded SBA-NH2 materials (Figure A3).

**Figure 4:**
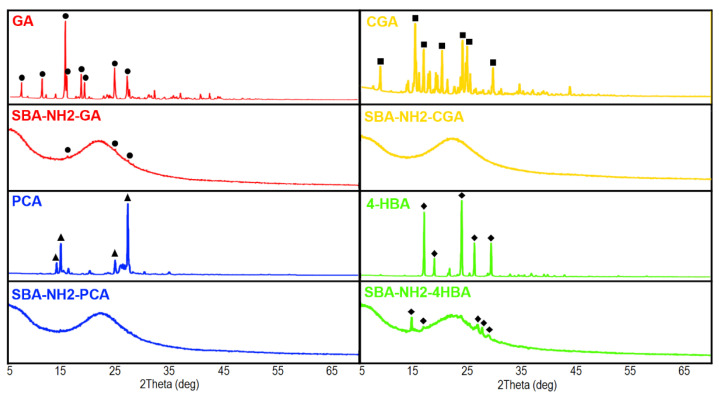
The XRD patterns of antioxidant-loaded SBA-NH2 materials with the antioxidant reference samples.

The XRD patterns of antioxidant-loaded SBA-NH2 materials together with antioxidant reference samples are presented in Figure 4. All antioxidants were characterized by sharp patterns with high intensity due to the fact of their crystalline structure. Samples of antioxidant-loaded SBA-NH2 materials revealed a broad halo in the 2-theta range of 15–30 degrees, which is characteristic of amorphous silica [63]. Highly reduced intensities of main peaks characteristic of GA were observed in the pattern of the SBA-NH2-GA sample. A similar observation was noticed for SBA-NH2-4HBA with additional shifts of peaks at 15 and 30 degrees. No peaks derived from CGA and PCA were detected in the XRD patterns of the SBA-NH2-CGA and SBA-NH2-PCA samples.

### 3.2. Desorption of Antioxidants and Antioxidant Capacity

The mass (m_Des_) and percentage (%Des) of antioxidants desorbed from the SBA-NH2 material (based on the HPLC method), together with the amount of desorbed antioxidants, with a preserved antioxidant capacity (FC method) and calculated antioxidant capacity (AC), are compared in Table 4. For GA, the %Des increased in the following order SBA-NH2-GA < MCM-41-GA < SBA-SH-GA < SBA-15-GA. In the case of SBA-NH2 silica material loaded with antioxidants, the highest %Des was observed for: PCA > CGA > 4-HBA and GA. The amounts of antioxidant desorbed, determined using the FC method, were approximately 1.2 ± 0.2 times higher compared to the HPLC method. Thus, it was assumed that all desorbed antioxidants preserved their AC.

## 4. Discussion

The highest m_Ads_ of GA was noticed for the SBA-NH2 material, which might be explained by the attractive forces between the positively charged amine group R-NH_3_^+^ on the silica surface and the negatively charged carboxyl group of GA, R-COO^−^. This phenomenon was also observed in a study performed by Wang et al. in which the m_Ads_ of tannic acid (polyphenolic compound of glucose and GA) increased for amine-functionalized MSMs compared to nonmodified silicas (500 and 4 mg/g, respectively) [64]. In our study, the content of GA in SBA-NH2-GA material was 12%; thus, it was in agreement with the GA content in MCM-41-NH2 silica (9.8%) as reported by Iraji et al., when GA was adsorbed from 10 mg/mL of ethanolic solution [45]. Moreover, the favorable interactions between carboxyl and amine groups were used to covalently graft GA onto various MSMs via the formation of amide bonds providing, i.e., 86.7 mg of GA content per each 1 g of SBA-15 material modified with (3-aminopropyl)triethoxysilane [44]. A lower %Ads of GA for MCM-41, SBA-15, and SBA-SH might be explained by the weaker interactions between the GA molecules and the silica surface. Both the hydroxyl groups and carboxyl of the GA could interact with surface silanol groups (Si-OH) via hydrogen bonding, which is much weaker than the ionic interaction between the GA and SBA-NH2 surface described above. Moreover, the ratio between the size of GA molecules and the pores’ diameter should also be considered. The computed average molecular diameter for GA is 0.8 nm [65]. Rashidi et al. found that an increase of the MCM-41 pore size from 2.4 to 3.4 nm enhanced GA loading two-fold [43]. Based on the obtained results (Table 1), the adsorption of GA onto SBA-15 (pore diameter: 6 nm) was lower by 30% than onto MCM-41 (pore diameter: ~2.5 nm). It might be assumed that a too high a ratio between GA molecule and pore size might reduce the %Ads. Similar findings were reported for the adsorption of drug molecules—higher drug loading was observed for MCM-41 despite its lower pore size in comparison to SBA-15 [56,66]. Moreover, the impact of different morphologies of MCM-41 (spherical particles) and SBA-15 (rod-like particles) on GA adsorption should also be taken under consideration and requires further studies.

The interactions between the amine-functionalized surface of SBA-NH2 and the carboxyl group of adsorbed CGA, PCA, and 4-HBA have also been confirmed using FTIR (Figure A2). The shifts of N-H bending vibrations characteristic of primary amines at 1540 cm^−1^ towards lower wavenumbers were observed in all investigated samples, proving the protonation of the 3-aminopropyl functional group. Moreover, to prove the abovementioned shifts in the N-H bands, we performed a preliminary study in which SBA-NH2 silica material was suspended in either 0.1 M HCl or 0.1 M NaOH solution for 24 h. The specific bands of protonated amines (N-H_3_^+^) were identified at 1501 cm^−1^ after soaking in 0.1 HCl, whereas N-H_2_ bending vibrations at 1537 cm^−1^ were observed for material soaked in 0.1 M NaOH (data not shown). Similar findings were reported by Richner and Puxty who proved the shifts of N-H bending vibrations towards lower wavenumbers during CO_2_ absorption by aqueous solutions of amines using in situ FTIR [67].

Among the four investigated antioxidants, the highest adsorption efficiency onto SBA-NH2 silica materials was noticed for CGA (290.8 ± 12.3 mg/g, Table 2). The main reason for this observation might be the differences in the chemical structures of the investigated antioxidants. Chlorogenic acid is an ester of two polyphenolic acids: caffeic and quinic acid; thus, it contains more hydroxyl groups compared to GA, PCA, and 4-HBA. Moreover, based on the obtained results, it could be observed that increasing the amount of hydroxyl groups in the antioxidant structure increased the %Ads (Table 2). Consequently, the %Ads increased in the order of 4-HBA < PCA < GA < CGA for structures with 1, 2, 3, or 5 hydroxyl groups, respectively. In a valuable study performed by Liu et al., the adsorption of different phenolic acids onto mesoporous silica materials functionalized with various amino-ligands was performed [68]. Similar to our results, CGA was characterized by higher adsorption performances compared to GA (i.e., 60 and 170 mg per 1 g of (3-aminopropyl)trimethoxysilane-modified silica, respectively). In the mentioned study, not only was the amount of hydroxyl groups considered but also the influence of pK_a_ values and geometry configuration. Nonetheless, the ionic interactions between the carboxyl group of antioxidants and the amine-functionalized surface of SBA-NH2 seemed to be dominant, which is why the adsorption efficiency among the four investigated materials (i.e., MCM-41, SBA-15, SBA-SH, and SBA-NH2) was the highest for SBA-NH2. 

Based on the TPM results (Table 3, Figure 3a), both the pore size and pore volume of antioxidant-loaded SBA-NH2 materials were lower than those of unloaded materials. The two-fold reduction in pore volume confirmed the adsorption of antioxidants and indicated their deposition inside the pores of the silica material. This is a well-known phenomenon observed in various studies. For example, Ravinayagam and Jermy adsorbed GA onto different types of MSMs. As a result, the loading of GA into the SBA-16-type silica caused a significant reduction in pore volume compared to the parent materials (from 0.49 to 0.27 cm^3^/g), suggesting favorable texture for pore filling and deposition of GA [69]. Herein, the reduction in pore size from 6 to 5 nm for parent and antioxidant-loaded SBA-NH2 silica material was observed, respectively. This may suggest that the blockage of small pores in the mesoporous structure occurred. Ionita et al. also observed the shifts in pore size towards lower values after the adsorption of resveratrol onto mesoporous silicas [41]. Moreover, the reduction in pore size after the adsorption procedure might be explained by the presence of some antioxidant particles precipitated on the silica surface (Figure 2), causing the partial blockage of the pore entrance.

The DSC thermographs (Figure 3b and Figure A3) confirmed the melting points of the reference antioxidants’ samples in agreement with the literature. However, peaks corresponding to melting points were not observed in antioxidant-loaded SBA-NH2 silica materials, which may suggest the existence of an adsorbate in an amorphous state. To further confirm this observation, we conducted an XRD analysis (Figure 4). Pure antioxidants were characterized by high crystallinity as indicated by the numerous sharp peaks presented in XRD patterns. For both PCA and GCA, antioxidant-loaded SBA-NH2 materials revealed no sharp peaks, which suggested the presence of these antioxidants in an amorphous form. For GA, the adsorption onto SBA-NH2 caused a significant reduction in the XRD peaks’ intensities, demonstrating the presence of GA inside the mesopores in a nearly amorphous state. Indeed, the adsorption of substances onto mesoporous silicas is one of the modern amorphization methods. Such observations have been reported not only for other antioxidants (such as resveratrol [41], quercetin [70], and morin [71]) but also for drug molecules adsorbed onto mesoporous silicas (such as fenofibrate [72], carbamazepine, and indapamide [73]). The capability of mesoporous silicas to suppress the crystallization of GA has been emphasized in other studies in which the GA-loaded mesoporous silica samples were characterized only by a broad halo of amorphous silica with no sharp peaks derived from GA [69,74]. Surprisingly, the XRD pattern of SBA-NH2-4HBA showed peaks of crystalline phase, which suggested the presence of 4-HBA rather in the semicrystalline form inside the mesopores. Additionally, some shifts in peaks’ positions were observed that might be connected with potential interactions between the silica surface and 4-HBA that induced several changes in the crystal structure of the antioxidant. 

Considering the HPLC desorption results (Table 4), the percentage of antioxidant desorbed from SBA-NH2 did not exceed 35%, excluding PCA for which the desorption was found to be 53.1 ± 0.5%. Interestingly, despite having only one hydroxyl group in its structure, 4-HBA had the lowest desorption efficiency, implying relatively strong adsorbate–adsorbate interactions. However, the interactions between the silica surface and the adsorbate are not the only factor that influences desorption. Other properties, such as antioxidant solubility or the presence of antioxidants in crystalline or amorphous forms, should also be considered. The amounts of antioxidants desorbed from SBA-NH2, determined using the FC method, were higher compared to the HPLC method (Table 4) which might be related to the different parameters of method validation. Nonetheless, it might be assumed that the total amount of antioxidants desorbed from all types of MSMs preserved their antioxidant capacity—the ratio of desorbed antioxidants with preserved antioxidant capacity (FC method) to the total amount of desorbed antioxidants (HPLC method) was higher than 1.0. In recent studies, the adsorption of antioxidants onto mesoporous silica is being performed to increase the stability of compounds and preserve their antioxidant capacity [69]. In our study, such an approach might be concluded for all of the investigated antioxidants as well.

## 5. Conclusions

Commercially available mesoporous silica materials may be considered as adsorbents suitable for the adsorption of gallic acid; however, the adsorption effectiveness varies and is determined by the type of adsorbent used. The highest adsorption of gallic acid was observed for amine-modified SBA-15 mesoporous silica, most likely due to the attractive forces between adsorbent and adsorbate. Amine-functionalized silica was characterized by satisfactory adsorption of other phenolic acids: chlorogenic, protocatechuic, and 4-hydroxybenzoic. The adsorption of antioxidants onto mesoporous silica caused their presence in the amorphous or semicrystalline form inside the pores. More importantly, different desorption effectiveness levels of the adsorbed compounds were observed with the antioxidant capacity preserved for all of the investigated antioxidants.

## Figures and Tables

**Table 1:** Mean adsorption efficiency together with the amount of GA adsorbed per 1 g of MSMs.

Material Type	m_Ads_ ± SD (mg/g)	%Ads ± SD
MCM-41	97.6 ± 27.5	18.8 ± 5.3
SBA-15	69.1 ± 7.8	13.3 ± 1.5
SBA-NH2	133.4 ± 27.8	25.7 ± 5.4
SBA-SH	67.9 ± 13.3	13.1 ± 2.6

**Table 2:** Mean adsorption efficiency together with the amount of antioxidants adsorbed per 1 g of MSMs.

Material Type	m_Ads_ ± SD (mg/g)	%Ads ± SD
SBA-NH2-GA	133.4 ± 27.8	25.7 ± 5.4
SBA-NH2-PCA	52.7 ± 13.5	8.1 ± 2.1
SBA-NH2-CGA	290.8 ± 12.3	55.2 ± 2.3
SBA-NH2-4HBA	54.5 ± 4.0	2.8 ± 0.2

**Table 3:** The diameter and pore volume of parent and antioxidant-loaded SBA-NH2 derived from H_2_O-TPM.

Material Type	D (nm)	V (cm^3^/g)
SBA-NH2	6.3	0.41
SBA-NH2-GA	5.2	0.21
SBA-NH2-PCA	5.0	0.16
SBA-NH2-CGA	below detection limit	below detection limit
SBA-NH2-4HBA	5.3	0.15

**Table 4:** The amount of antioxidants desorbed from the SBA-NH2 material determined using the HPLC and FC methods with antioxidant capacity.

Material Type	HPLC		FC		AC
	m_Des_ ± SD (mg)	%Des ± SD	m_Des_ ± SD (mg)	%Des ± SD	
MCM-41-GA	1.5 ± 0.02	31.2 ± 0.5	1.9 ± 0.09	39.2 ± 1.8	1.3
SBA-15-GA	1.7 ± 0.04	58.2 ± 7.7	2.21 ± 0.06	77.0 ± 10.0	1.3
SBA-15-SH	1.3 ± 0.06	39.1 ±1.8	1.58 ± 0.06	46.4 ± 1.6	1.2
SBA-NH2-GA	1.8 ± 0.02	27.4 ± 0.2	2.3 ± 0.1	35.1 ± 2.2	1.3
SBA-NH2-PCA	1.4 ± 0.01	53.1 ± 0.5	1.4 ± 0.06	52.4 ± 2.3	1.0
SBA-NH2-CGA	5.0 ± 0.14	33.8 ± 0.9	5.3 ± 0.05	35.4 ± 0.3	1.1
SBA-NH2-4HBA	0.71 ± 0.03	25.9 ± 1.1	1.04 ± 0.09	38.0 ± 3.3	1.5

## Data Availability

The data are contained within the article.
